# Towards future-oriented conservation: Managing protected areas in an era of climate change

**DOI:** 10.1007/s13280-018-1121-0

**Published:** 2018-11-17

**Authors:** Lorrae van Kerkhoff, Claudia Munera, Nigel Dudley, Oscar Guevara, Carina Wyborn, Carolina Figueroa, Michael Dunlop, Melissa Abud Hoyos, Javier Castiblanco, Laura Becerra

**Affiliations:** 1 Fenner School of Environment and Society, The Australian National University, Acton, ACT 2601 Australia; 2 Equilibrium Research, 47 The Quays, Cumberland Road, Spike Island, Bristol, BS1 6UQ UK; 3 World Wildlife Fund Colombia, Oficina Principal, Carrera 35 No. 4A-25, Cali, Colombia; 4 Luc Hoffmann Institute, IUCN Conservation Centre, Rue Mauverney 28, 1196 Gland, Switzerland; 5 Department of Society and Conservation, University of Montana, 32 Campus Drive, Missoula, MT 59801 USA; 6 Commonwealth Scientific and Industrial Research Organisation (CSIRO) Land and Water, GPO Box 1700, Canberra, ACT 2601 Australia

**Keywords:** Climate adaptation, Colombia, Conservation governance, Ecological transformation, Futures thinking, Science–policy interface

## Abstract

Management of protected areas must adapt to climate impacts, and prepare for ongoing ecological transformation. Future-Proofing Conservation is a dialogue-based, multi-stakeholder learning process that supports conservation managers to consider the implications of climate change for governance and management. It takes participants through a series of conceptual transitions to identify new management options that are robust to a range of possible biophysical futures, and steps that they can take now to prepare for ecological transformation. We outline the Future-Proofing Conservation process, and demonstrate its application in a pilot programme in Colombia. This process can be applied and adapted to a wide range of climate adaptation contexts, to support practitioners in developing positive ways forward for management and decision-making. By acknowledging scientific uncertainty, considering social values, and rethinking the rules that shape conservation governance, participants can identify new strategies towards “future-oriented conservation” over the long term.

## Introduction

Conservation managers in the twenty-first century are confronting relentlessly increasing pressure to cope with change. The magnitude and nature of potential future ecological transformation challenges the very foundations of conservation (Stein et al. 2013; Wyborn et al. 2016). The most commonly accepted norm for dealing with this pressure is to seek out scientific and technical advice for decision-making, including species adaptiveness (Beever et al. 2016), ecological modelling (Hannah et al. 2017), vulnerability (Metcalf et al. 2015) and projected changes (Foden et al. 2013). As West et al. wrote, the idea of “managing for change” in conservation “…will be a challenging proposition since it is difficult to anticipate threshold changes and because the array of potential states into which a system may change is highly uncertain; thus concentrated research to understand the characteristics and indicators of threshold responses will be essential” (West et al. 2009, p. 1018). Yet despite the prevalence of this norm, there is growing awareness that there may be limits to technical solutions, as climate adaptation is recognised as a complex socio-political process involving authority, knowledge and subjective values (Hagerman et al. 2010; Lemieux and Scott 2011; Eriksen et al. 2015). Over the last decade, a growing array of scholars are drawing attention to the institutional dimensions of climate adaptation in conservation. Rannow et al. (2014) highlight technical and legal issues for protected areas, while Abrahms et al. (2017) make recommendations on technical knowledge, tools and frameworks for managing protected areas under climate change. Yet despite established frameworks and principles (Gross et al. 2016), analysts note that “Implementation of adaptation plans and strategies continues to lag…” (Stein et al. 2013, p. 2, see also Jantarasami et al. 2010; Wise et al. 2014). In general terms, this can be regarded as the fault line that emerges when new (climate adaptation) demands are imposed on organisational structures that have been created and evolved to meet the old (maintain existing ecosystems) demands. As Stein et al. (2013) go on to observe “…while the concept of adaptive capacity is often thought of in reference to the species and ecosystems that are the targets of adaptation action, the ability of institutions themselves to adjust and evolve will be key to their ability to change” (p. 508). (See also Dunlop et al. 2013; Wyborn et al. 2016.) But although there is growing documentation of *what* conservation institutions should do to govern for climate adaptation, relatively few have asked *how* they can adjust or, as Pelling (2011) argues, transform in the face of climate change *from their existing governance structures*?

This article reports on a project that sought to approach this question by working with organisations at the forefront of conservation governance practice. The Future-Proofing Conservation project was developed to explore new ways of managing protected areas when there is potential for large-scale, rapid and transformative ecological change. Drawing on Pelling et al.’s (2015, see also 2011) differentiation between transitions: “incremental adjustments that preserve systems integrity when conditions change” and transformation: “measures that challenge the stability of current systems” (p. 116), we sought to establish processes and tools that enabled transitions from traditional approaches to ‘future-oriented conservation’, and to investigate whether incremental transitions could accumulate to larger transformations. This project was formed by a partnership between researchers (The Australian National University, the Luc Hoffmann Institute, The Commonwealth Scientific and Industrial Research Organisation), civil society (World Wildlife Fund Colombia, WWF-C), practitioners (Parques Nacionales Naturales de Colombia, PNN), and conservation advisers (Equilibrium Research). (Throughout this article we refer to the core collaborative research team members as the ‘research team’ (authors), a broader group of engaged policy and management colleagues from WWF and PNN as ‘partners’ and practitioners who participated in activities as ‘participants’.) Collectively, we developed an approach to enabling transitions towards more “future-oriented” conservation (Wyborn et al. 2016). The Future-Proofing Conservation process that emerged is a structured, interactive, dialogue-based series of activities. These activities encourage conservation practitioners to consider their current management approaches in light of future climate change, and to explore alternatives based on diverse social values and benefits. In this article, we present the Future-Proofing Conservation process, illustrated by pilot studies in Colombia.

## Conceptual framework for future-oriented conservation

The Future-Proofing Conservation project was founded on a philosophical perspective drawn from evolutionary learning. Ansell and colleagues (Ansell and Gash 2007; Ansell 2011) propose that evolutionary learning takes place through three central and interconnected activities. First, taking a problem-driven perspective, ensuring that abstract concepts (in our case, around futures thinking, transformation, governance and values) are grounded in actual problems and the experience of participants, and a thorough understanding of decision-making contexts. Our collaboration directly involved practitioner partners to develop shared understanding of climate adaptation as a management problem and its socio-political framing. Second, it recognises that being reflexive about individual and institutional frameworks which shape possible actions can create new options for change. We considered both the formal institutional arrangements and the historically embedded informal expectations and norms that influence conservation actions (Ostrom 1999). Third, it involves creating spaces where stakeholders can develop ideas, discuss social values, share information, and consider current institutional rules and future possibilities for change. We co-designed processes that encourage partners and participants to work together to explore implications of new information and create new understandings and solutions.

Within this broad philosophical framing we drew on the conceptual heuristic of values-rules-knowledge (VRK) as developed by Gorddard et al. (2016) as an accessible way to engage with the contextual factors shaping how our partners and participants approach climate adaptation and conservation. VRK emphasises that climate adaptation in conservation can be enabled not only by updating or increasing what we know (technical knowledge); it also requires changing the rules that shape policy and practice (governance and management); and evolution of values (socio-political preferences) driving conservation. Addressed separately, they can each support incremental change. But deliberately driving co-evolution of values, rules and knowledge together can lead to a more meaningful transformation in thinking and action towards the goal of “future-oriented conservation”.

### Four transitions towards future-oriented conservation

We define future-oriented conservation as policy, planning and management that effectively anticipates change and actively prepares for it over the long term. In order to achieve this, there are currently a wide range of possible approaches, tools, methods, strategies and processes (see Gross et al. 2016; Abrahms et al. 2017). For protected area policymakers, planners and managers, it can be difficult to know where to start, especially when tools or methods have been developed to suit other ecological, social and political contexts, and different possible futures. We distilled from the literature four propositions regarding how future-oriented conservation could be achieved via four transitions for conservation managers:

1. *From strategies and practices based on resisting ecological change to strategies and practices that anticipate and accommodate ecological change* Conservation is, almost by definition, concerned with conserving ecosystems, species or landscapes in their current state or restoring them to a previous state. Related climate adaptation literature and scientific research more generally have tended to frame the primary goal of adaptation as building resilience in ecosystems, increasing the capacity of ecosystems to maintain or return to their core characteristics despite changing climatic conditions. However, strategies based on resilience, stasis or restoration may not be sufficient in a changing climate (Baron et al. 2009; West et al. 2009; Boyd et al. 2015), and may even be counterproductive if resources are directed towards maintaining features that will inevitably change (Dunlop 2013). Anticipating and accommodating involves both practical changes and a more conceptual change, as traditional conservation ideals, goals and targets (key species, historic communities) might no longer be suitable guides for management (Tschakert and Dietrich 2010). This is not simply substituting one source of information for another, but a shift in the social and political basis of decision-making (Adger et al. 2009), from objective “things” (species, ecosystems) that were previously central to conservation concern to subjective “values” that are contestable and more overtly political.
2. *From conservation goals and targets focusing on ecological attributes to goals and targets that also focus on social values and benefits* Conservation goals that relate to specific attributes such as particular species or communities may become unreachable as ecosystems change. While including social values is sometimes noted as an important aspect of adaptive management for protected areas (e.g., Lockwood 2006; Tanner-McAllister et al. 2017), we propose that a focus on social values and benefits can offer an additional management path, as benefits may persist even as ecological attributes change. For example, where an important benefit of a protected area is supply of clean water, the landscape processes that produce clean water may remain even if the species of trees or understory in a forest change. This helps people to understand that benefits can persist even if ecosystems transform. For simplicity, we deliberately merged *benefits* (what people get) and *values* (what people like/want) by assuming that people value something that provides benefits. This transition implies a general recognition that maintaining benefits experienced by people may be different from maintaining the current state of the ecosystems themselves (Chan et al. 2006).
3. *From understanding climate adaptation as a scientific issue to understanding it as a governance issue* Scientific knowledge and technical solutions are often assumed to be the best resources and strategies to support conservation (Godet 2006; Gabriel 2014; Rannow et al. 2014), and many partners and participants in our study reflected this view. This is consistent with Archie et al’s empirical research where conservation decision-makers noted “lack of information” as a key barrier to adaptation (Archie et al. 2012; see also Lonsdale et al. 2017). While climate change projections are improving, the impacts on specific protected areas are typically uncertain, and implementation options implied by technical analysis can be infeasible (Lemieux and Scott 2011). Yet even with precise information, managers are confronted by a complex array of social and political challenges when making adaptation decisions that emerge from contested values, multi-layered policy challenges and institutions that are often resistant to change (Eriksen et al. 2015). Focusing on climate adaptation as a governance issue opens conversations about how we organise ourselves to address changing ecosystems, including identifying other (non-technical) information that may also be useful for decision-making. For example, incorporating social values and benefits requires consultation on what those benefits are, who receives them and how they are prioritised.
4. *From conservation practices based on problem-solving to practices based on ongoing learning* All of the above imply a more dynamic and uncertain management environment; the problem of climate change cannot be “solved” in any conventional sense. Consistent with adaptive management approaches, interventions need to be informed by new knowledge from many sources, and adapt as biophysical conditions and societal values continue to change (Olsson et al. 2006; Berkes et al. 2008). Research on anticipatory approaches suggests management should complement activities focused on solving defined problems (e.g., a water shortage) with learning as social and ecological conditions change (e.g., water planning) (Tschakert and Dietrich 2010; Boyd et al. 2015).

**Fig. 1:**
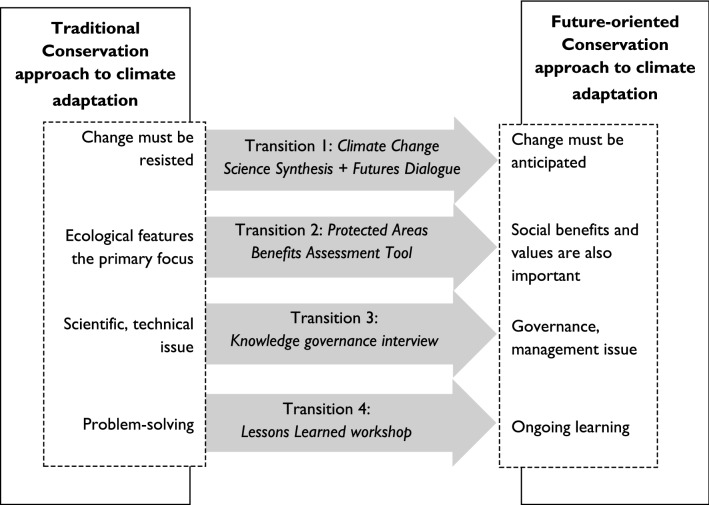
Four conceptual transitions and the tools that support them, that accumulate to the larger transformation, from traditional conservation thinking and practices towards future-oriented conservation

These propositions are illustrated in Fig. 1.

In the diagram, we recognise that “Traditional Conservation approach to climate adaptation” is a stereotype, as most conservation practitioners are at least part way along at least some of these transitions. Activities in italics in Fig. 1 were designed to determine where participants were between the “traditional” and the “future-oriented” approaches, and to develop a shared conceptual base from which we could explore possibilities for transforming conservation management. For example, in Colombia, protected area managers and conservation agencies were well advanced in understanding the need for different approaches to management (Transition #1) and were part way towards creating a learning-based approach (Transition #3), which generated opportunities to advance the other transitions.

These transitions offer useful entry points into the complex and difficult questions of how social, political, economic and cultural forces form the context for adaptation decision-making, and were used throughout the process to help navigate the range of options and guide the development process. Importantly, they were regarded as cumulative, rather than independent—we were not looking to choose between them but to find context-relevant tools and processes that could advance all of them. Pelling (2011) differentiates between resistance that seeks to maintain existing structures and processes; transitions that represented incremental changes that do not challenge existing social and political structures; and transformations which represented change in political regimes. Our overarching proposition is that individually these propositions are incremental transitions, but cumulatively they can support a transformative change in social and political organisation.

## Materials and methods

### Case study: Protected areas management in Colombia

Colombia is one of the most biologically diverse countries in the world, with over 9000 endemic species; and habitats that vary from Andean glaciers to tropical rainforests and arid deserts. Culturally, Colombia is home to over 100 different ethnicities, including pre-Colombian indigenous peoples and Afro-Colombian communities. This diversity is represented in 59 natural areas (2017) that belong to the Systems of National Natural Parks (SINAP), which represent 14 268 224 ha; 11% of continental land surface and 1.5% of marine jurisdiction. This is a multilevel system that provides managerial instruments to stakeholders from different governance sectors and levels (private, communitarian, regional, national scale) for terrestrial and marine areas within broader “strategic landscapes”, providing specific rules and policies for their implementation (CONPES 3680, law 2372 from 2010). PNN is a Special Administrative Unit with administrative and financial autonomy and jurisdiction in all the national system of protected areas.

Climate adaptation has appeared in Colombia´s protected areas portfolio since 2004, acknowledging climate as major driver of transformation in landscapes and biodiversity. From the outset, conservation–based actions were identified as a key component of the country’s climate adaptation agenda. Adaptation work in Colombia aimed to provide evidence about the role ecosystems play in enabling people to adapt and for mobilising resources for conservation. By doing so, it also informs and influences decisions towards vulnerable people, places and ecosystems, and ensures that the conservation of priority places and ecosystems is incorporated into the ongoing climate and development planning processes. WWF-C has taken a national leadership role on these issues. Colombia was selected as our case study due to WWF-C’s role in developing “Climate Smart Conservation”, a learning-oriented approach based on anticipation of climate change, and PNN’s work understanding the impacts of climate change and developing associated policy initiatives. The Future-Proofing Conservation process was piloted in two areas where WWF-C work closely with PNN: Amazon Piedmont (Churumbelos, Guacharos and Alto Fragua National Parks), and the Coffee-Growing Region (Otun Quimbaya Flora and Fauna Sanctuary and Nevados National Park). These sites were selected as presenting diverse contexts (biophysically and socially) as well as diverse histories of engagement with climate adaptation.

The Future-Proofing Conservation project was composed of two phases. First, a co-design and co-production phase, where the research team worked closely with a small number of partners from PNN to develop a context-relevant intervention that could assist a wider range of participants (protected area managers and policymakers) engage with the transitions described in “Four transitions towards future-oriented conservation”. The activities in each phase are summarised in Table 1. Second, a piloting phase, where the draft intervention was tested in two pilot protected areas. While the main focus of this article is to document the implementation in Phase 2, a brief description of Phase 1 will be given here.

**Table 1:** Summary of activities in Phase 1 and Phase 2 of the project

Phase	Phase 1: Co-design and co-production				Phase 2: Piloting future-proofing conservation		
Date	August 2015	Nov 2015	May 2016	October 2016	March–April 2017	April 2017	May–June 2017
	Initial scoping workshop	First co-design workshop	Second co-design workshop	Third co-design workshop	Pilot phase planning and implementation	Pilot phase site #1 Amazon	Pilot phase site #2 Otun
Duration	3 days	2 days + team debrief	2.5 days + debrief	2.5 days + debrief	Ongoing	2 days	2 days
Participants	International participants: academics (3), civil society (15), practitioners (2)	Research team and core partner representatives: academics (5), civil society (9), advisers (1) and practitioners (15)	Research team and core partner representatives: academics (4), advisers (1) civil society (7), and practitioners (5)	Research team and core partner representatives: academics (3), advisers (2) and civil society (7) and practitioners (3)	WWF team and partner representatives (various) from Pilot site #1, plus community representatives for PA-BAT activity	Research team and participants from Pilot site #1: 14 Park managers, 2 Territorial (provincial) staff, 3 National office staff	WWF team and participants from pilot site #2
Aim	Generating ideas and critical input for building a ‘ground up’ approach to FOC	Learning from partners about PNN context. Establish a shared vision for the project. Test initial ideas for concepts and methods	Learning from partners about PNN context. Testing refined ideas for concepts and methods	Test “futures workshop”. Refine ideas consolidated into a proposed multi-step process for staff engagement	Test “Lessons learned” and PA-BAT activities; collect data regarding knowledge and decision-making	Test the Futures Dialogue final workshop of the proposed process	Test the full process
Activities	Brainstorming, theory of change, small and large group discussion	Presentation, feedback and discussion. Additional research needs identified	Presentation, feedback and discussion. Pilot sites identified	Feedback and discussion, further refinement and planning for piloting	Lessons learned workshop; PA-BAT workshop; knowledge governance interviews	Structured discussion based on the questions of the FD framework. Feedback and lessons	Lessons learned, PA-BAT, Futures Dialogue workshops. Feedback and lessons
Facilitation	Facilitated by academic partners	Facilitated by academic partners and WWF	Facilitated by academic partners and WWF	Facilitated by WWF	Workshops facilitated by WWF. Interviews conducted by academic partners	Facilitated by WWF. Presentations by academic partners	Facilitated and presented by WWF

### Phase 1—Co-design and co-production

The aim of the first phase of the project (2015–2017) was to develop an interactive process that could be applied by PNN or WWF partners to support the transitions towards future-oriented conservation in their organisations. In accordance with our evolutionary learning approach, Phase 1 drew on design thinking (Brown 2008; Adams et al. 2011; Beier et al. 2016) and co-production processes (Kirchhoff et al. 2013; Mauser et al. 2013; Clark et al. 2016) to collaboratively explore the challenge in context; to generate shared visions for the future; and to create new ideas for achieving the transitions described in “Four transitions towards future-oriented conservation”. The research team worked with partners to learn about the context that policymakers and protected area managers operate within, establish shared goals, and test workshop activities and facilitation processes. Phase 1 included four workshops organised from August 2015 to June 2017. The participants, aims, methods and leaders are summarised in Table 1. In these workshops, the academic research team presented tools or concepts from the literature, which were tested and critiqued by partners and practitioners, and discussed extensively. The heuristic of VRK was applied throughout to ensure that the conversation included deliberations on social and political values; institutional settings, conventions and rules; and technical and non-technical knowledge. Formal and informal feedback processes, theory of change (Vogel 2012), and scorecard evaluations were used to provide multiple avenues of dialogue and reflection, and document the process as it advanced.

**Table 2:** Summary of results and outcomes from the pilot workshops

Activity	Results Amazon	Results Otun	Outcomes	Feedback
PA-BAT workshop	PA main benefits identified, in order of priority: Reducing climate change impacts Water quality, quantity and access Sacred places/sites Tourism	PA main benefits identified, in order of priority: Water quality and quantity Sites of unique beauty Reducing climate change impacts Tourism	Communities and PA staff generated shared documentation of priority benefits, ranked in order of importance. Additional outputs such as maps included specific locations of sacred sites	Participants Pilot #2 “The tool is useful to engage diverse stakeholders; as there are no ‘wrong’ answers, all can participate. Wider representation of other community groups would have improved the workshop”
Lessons learned workshop	Participants noted that: Risk management is linked to climate change Little monitoring and research completed, what has been done is uncoordinated Communities are aware of climate change; opportunities for education	Participants noted that: Technical studies of climate change impacts have been completed Limited capacity to develop management changes based on research Solid foundation of cooperation across sectors and communities	Barriers identified: Institutional complexity and disconnect Poor knowledge sharing Existing knowledge is too technical for many local managers Local knowledge is not recognised	Participant Pilot #1 “Climate is not something that depends on a specific space, we must expand … and link ourselves to a larger scale than the protected area”
Futures dialogue	Participants identified actions that could be taken now to prepare for climate change, including Extending networks for collaboration in planning, such as water utility departments; Reviewing existing and potentially establishing new monitoring programmes to document baselines for benefits as well as species; Integrating PABAT into existing planning frameworks to ensure benefits are considered alongside conventional objectives	Participants identified actions, recognising that *transformation* is different from *impacts,* including Need to review conservation objectives in light of transformation; Protected areas alone cannot ‘save’ biodiversity, must involve communities; Larger scale (territorial) organisation and planning is needed, but difficult	Participants developed an alternate way of thinking about planning for climate change that reflected the current state of technical knowledge, incorporated values and benefits, and considered existing decision-making processes and tools. Participants did not follow through the change in thinking with a fully revised approach to PA policy, planning and management; ideas were generated but proposed incremental rather than transformative changes.	Participant FD #1 Park manager “We need to manage relationships with other actors, to maintain benefits that Parks generate” Participant FD #1 National office: “This project has achieved a dialogue between different [park management] levels”. Participant FD#1 Park manager: “how do we take these ideas into policies, plans and management frameworks for conservation?”

Following each workshop, the research team distilled the feedback and gradually refined the approach to a suite of concepts and tools that became the “Future-Proofing Conservation process”. Key issues that emerged from Phase 1 included a need to synthesise and summarise existing technical knowledge on climate change relevant to the pilot sites; more intensive research to understand the complex decision-making settings within PNN; the value of opportunities to reflect on previous work and existing knowledge in protected area sites; and activities to engage communities as well as professionals in identifying opportunities for change.

### Phase 2—Piloting the Future-Proofing Conservation process

The Future-Proofing Conservation process that emerged from Phase 1 comprises a tailored technical synthesis report, interview-based research, 2 preliminary workshops, and a final culminating workshop. A summary of the process, results and outcomes are presented in Table 2.

#### Climate change science synthesis report

Ability to examine and explore locally relevant information on ecological impacts of climate change was an important initial stage for participants in the process. This was both to inform decisions, and to provide reassurance that science was ‘covered’ before focusing on management. Thus, we recruited a local expert (author, Abud Hoyos) to work with the academic team to create a synthesis report focused on the pilot areas. This brought together the best available projections and expected ecological change in the specific sites.

The synthesis focused on documenting diverse observed and anticipated impacts on biodiversity and ecosystem services, not just expected species movements or loss, and drew on modelling to indicate the potential magnitude of change. Expected impacts included melting glaciers, changes in water provision, increased coastal erosion, shifts in species distribution and migration patterns, more extreme climatic events, and changes in ecosystem types and function. Changes in species and ecosystems in protected areas affect the provision of ecosystem benefits, and other threats to biodiversity may be exacerbated. For example, projections show that warmer temperatures may force coffee growers to move crops to higher altitudes. Impacts may be amplified by existing threats such as invasive species, habitat loss and fragmentation, mining, agricultural and urban expansion. Many questions remain unanswered, and discussion in the synthesis report explicitly considered the high level of uncertainty but also highlighted impacts that are likely.

By emphasising both what we do know as well as the limitations of the current state of knowledge, participants were confident that they were not deliberating management or governance in ignorance of the scientific knowledge base. Emphasising the pervasive and inevitable nature of ecological change underpinned Transition #1, from resisting change to anticipating it. The synthesis report was incorporated into the final stage of the Future-Proofing Conservation process, the Futures Dialogue workshop.

#### Knowledge governance mapping

Knowledge governance mapping is an interview-based method where key stakeholders share their experience of knowledge-based processes and the application of climate information to decisions within their social and institutional context (van Kerkhoff and Pilbeam 2017). These interviews provided in-depth understanding of existing governance structures, supporting Transition #3: from climate change as a scientific issue to a governance issue. We conducted semi-structured interviews with 28 participants with an active role in protected areas planning and management to understand how scientific information (especially climate and ecosystem services) influenced decision-making and management of protected areas. Participants were purposively sampled to include managers and practitioners from national, territorial and local levels at the pilot study sites. Participants were encouraged to reflect on whether, how and to what extent climate adaptation is understood to be a scientific issue and/or a governance one, and to gauge how well these two domains were connected. Interviews were digitally recorded, transcribed, and thematically analysed.

The interviews revealed that protected area managers and agencies have been actively looking for information to support decision-making regarding management of biodiversity under climate change, and so were largely at the Traditional side of Transition #3. Barriers to moving towards a governance-based approach included the perceived need to build a shared understanding of climate variability, ecological transformation and climate change. Participants also identified the need for clarity on key management questions before they could use climate information strategically, grappling with how climate adaptation might ‘fit’ within existing structures. The findings from these interviews were used as background for the research team to inform the interactive sessions; they were also summarised in the Futures Dialogue (“Futures Dialogue workshop”) to encourage deliberation on existing governance structures.

#### “Climate adaptation lessons learned” workshop

This workshop compiled experience on what has previously been learned about climate adaptation from projects, policies or planning activities, addressing Transition #4: problem-solving to ongoing learning. This acknowledges that participants are not ‘starting from zero’, but have relevant expertise and experience and that understanding the adaptation context requires an assessment of local knowledge and perspectives. The workshop engaged local managers at the pilot sites. The objectives were to: (i) understand whether, how and to what extent local PA staff have integrated climate change into management strategies; (ii) understand opportunities and barriers for climate change management on a PA level; and (iii) create a space for sharing knowledge on climate change and PA management. The workshops sought to meet the need identified in the interviews by creating a shared understanding of key climate concepts and practices (known in Colombia as “conceptual levelling”); exploring participants perceptions of climate-related impacts; and creating a learning journey, a shared timeline of climate adaptation-related activities, policy changes or other significant developments. Through these activities, participants reflected on their existing expertise, their observations, and on processes of change including what enabled or prevented further developments, and associated challenges and opportunities. Different interpretations of events were explored and there was no pressure to reach a single narrative. The output of the learning journey was a large visual timeline that documented the evolution of climate change thinking and action, with the most significant lessons drawn from this experience.

This activity enabled collective reflection on processes of change at the site level. The research team was interested to see whether previous actions focused on discrete projects or tackled more systemic, interconnected issues. Where activities addressed knowledge generation, we looked both at the products (such as climate projection maps) and the processes through which these informed policy, planning or management (facilitating learning).

#### Protected areas benefits assessment workshop

Following from the “Lessons learned” activity, we used the Protected Area Benefits Assessment Tool (PA-BAT) to identify the benefits derived from a PA, creating the groundwork for Transition #2: from ecological attributes to social values and benefits (extended in the Futures Dialogue, see “Futures Dialogue workshop”). PA-BAT provides a quick and standardised way of collecting information about the ecosystem services derived from a protected area, drawing on knowledge from a range of stakeholders (Dudley and Stolton 2009). It is an inclusive, workshop-based approach that assembles a diverse group of stakeholders and reaches decisions by consensus. In this context, the workshops included local level PA managers (most of whom had participated in the previous workshop) but also included local community representatives and stakeholders.

The PA-BAT consists of 24 questions covering different benefits related to provisioning services (food, water, materials), regulation services (climate risk reduction, natural hazards control), cultural services (tourism, recreation, aesthetic values, education, knowledge generation, cultural and historical values, mental wellbeing) and regulation services (pollination, pests control). Participants explore current and potential importance (economic and non-economic) and the critical question of who benefits as well as location in the protected area (Dudley and Stolton 2009). Prior to the workshop, managers and others identify which benefits are to be assessed (the tool is global so not all will be appropriate) and approach different stakeholders (15–30 people) to participate. A series of slides are projected to summarise the key benefits through photographs and words in the local language. These slides allow discussion about the importance (economic and non-economic) and potential of the benefit. While based around a set series of questions, facilitators are open to other benefits and oral testimony is recorded; these stories are often the most useful part of the exercise. In Colombia, we employed an artist to summarise perceptions of the benefits and linkages, using a combination of sketches, icons and words. Participants were also invited to locate some of the benefits directly onto maps, with debate and discussion over exact locations revealing benefits which were often unknown by the management. PNN staff in Colombia also argued strongly that participants should be given the opportunity to discuss illegal but common uses as well as part of a separate but linked process (Figueroa and Behar 2017).

The PA-BAT was implemented in Colombia in the two pilot areas. For Otun, a landscape of protected areas in the Andes mountains, water quantity and quality, places with unique beauty, and reducing climate change effects such as heatwaves or drought, are perceived as the most important benefits of conservation. For the more remote Amazon Piedmont protected areas, knowledge generation, reducing climate change effects and tourism were perceived as the most important benefits. This set the stage for considering the relationships between ecological features and social benefits, and so ‘priming’ participants for the Futures Dialogue (“Futures dialogue workshop”). It also helped facilitators to gauge how readily participants can identify benefits that are connected with, but separate from ecological features, as a key component of Transition #2.

#### Futures Dialogue workshop

All of the previous activities established the basis for the final step, the Futures Dialogue workshop. This 2-day workshop guides participants through a series of structured questions, working through the four transitions to reach the final goal of defining practical steps towards governance that effectively anticipates climate change and prepares for it. The questions are summarised in Table 3, which also indicates the dominant concept and supporting resources from previous activities that were used in each step. In between interactive sessions, members of the research team gave short summary presentations relating to the previous activities, as a reminder and in recognition of the fact that not all participants had attended all activities. Participants are arranged into small groups of 6–8, and a facilitator at each small group guides the conversations.

**Table 3:** The question-based structure of the Futures Dialogue, with links to core concepts (column 2) and prior activities (column 3)

Stage 1: Values, benefits and transformation		
Question	Focus on	Resources
A—What benefits are provided by the protected area?	Values	PABAT
B—What ecological or landscape features provide that benefit?	Knowledge	Climate change synthesis
C—How might that feature transform under climate change?	Values and knowledge	PABAT, CCS
D—as the climate changes, what features will remain or stay the same?	Knowledge	CCS
E—How might the benefit be affected by climate-related change?	Values and knowledge	PABAT, CCS
F—what could be done differently to maintain benefits as change occurs?	VRK	All
Stage 2: Managing differently		
G—what would managers and planners need to do better/more to maintain benefits? What would need to be done differently? Should priorities change?	Rules, values	Lessons learnt
H—what are the barriers and opportunities for change?	VRK	Governance mapping
I–J—who is responsible for change? Who has authority to change?	Rules	Governance mapping
K—how can we prepare our management system for change? How do we engage those with authority to learn about multiple implications and anticipate change?	VRK	All

The first sessions aim to build shared understanding that *ecological change is inevitable* and recognise the difference between ecological change as a biophysical phenomenon and its effects on benefits for society. Questions A-B draw linkages between ecological features and benefits. Facilitators start with values participants expressed through the previous PA-BAT workshop as benefits from protected areas, and consider whether the ecological or landscape features that support that benefit are known to be vulnerable to change.

The participants then differentiate climate-related ecological change and potential changes to benefits, recognising that benefits will not necessarily be affected directly or proportionately (questions C–E). Through facilitated discussion the groups identify changes in management that could help maintain benefits in the longer term (questions F, G), and near-term opportunities and barriers, including knowledge gaps, to achieving those changes (question H). This includes recognising that access to detailed scientific information is not always essential to start planning for climate change adaptation. Finally, participants considered the process of changing management more specifically: what would need to change and how, and the people, organisations or institutions that can help enable these changes (questions I–K). This session includes consideration of incremental changes to implement now, and larger changes needing individual and institutional learning, political groundwork and ‘windows of opportunity’ before more systemic transitions could be tackled.

For example, in the Otun Coffee-Growing Region, participants identified “Livelihoods from tourism” as an important benefit. The questions guided participants to recognise that tourism is not solely dependent on particular ecological features, but can be supported in a changing environment. This may, however, require different or additional management to support the provision of benefits. The Futures Dialogue questions stimulated discussion and insight; however, while the first few questions were easier to address, the later questions needed more guidance and a greater level of facilitation.

## Results: The Future-Proofing Conservation process

While the Future-Proofing Conservation process emerged from Phase 1 and so is, in itself, a ‘result’, in this section we will summarise some of the main outcomes from the three workshops (Table 2). Given the multi-step process it is not possible to give a detailed account of results from all activities. We will focus on the three workshops as they comprised the ‘interventions’ towards the transitions. The climate synthesis report and knowledge governance interviews are reported elsewhere.

Feedback from participants suggests that that the process was successful in ‘reframing’ climate adaptation from a primary focus on protecting ecological characteristics to explicitly include societal benefits, and understanding climate adaptation as a management issue. Participants appreciated that the process enabled them to think deeply about the ‘bigger picture’ of conservation, and relate that to their more immediate management context. The process helped to increase agency of some participants who had previously felt powerless to enact change and fostered a shared sense of responsibility. The final part of the Futures Dialogue, exploring the strategies that participants could readily implement to enable learning-based conservation governance, was the most challenging part of the process. Future applications of the process will allow participants more time to explore the practical implications and develop action plans. PNN have already integrated the PA-BAT into their stakeholder consultation processes, as they recognised the value of identifying the benefits local communities can gain from protected areas as having multiple implications for their management. The Future-Proofing Conservation process is being adapted and developed further within WWF-C to inform new projects.

**Fig. 2:**
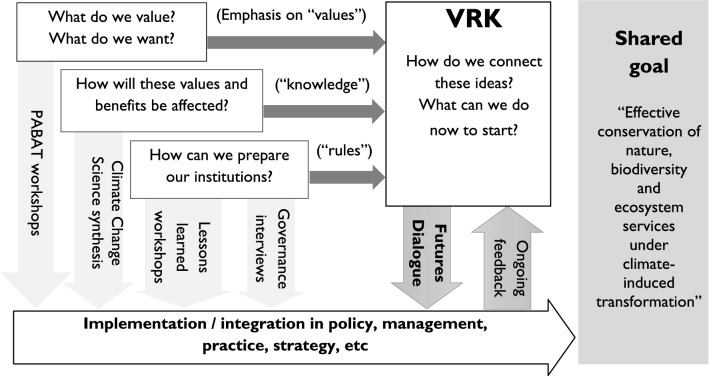
The Future-Proofing Conservation process, where activities (vertical arrows) connect concepts and practices towards the shared goal

Figure 2 illustrates how the different activities came together to support this transformation goal. This final structure could be applied across range of contexts for enabling new ways of thinking about climate adaptation and conservation; and as a basis for identifying new options for preparing for ecological transformation.

While each of the lead up activities played important roles in establishing the foundations for the Futures Dialogue, this final activity was a powerful way to integrate the emerging insights into a more consolidated understanding of future-oriented conservation.

## Discussion: towards future-oriented conservation

Future-Proofing Conservation aims to enable the transformation of conservation management to better accommodate uncertain, but different, climate-affected futures. We were responding to the gap noted in “Introduction”, that despite a substantial array of guidance, toolkits, and principles for *what* managers should do, a lack of action and implementation remains. In this project, our partners were, from the outset, interested and willing to explore the idea that transformations in the way protected areas were governed may be necessary. We were, then, collectively confronting the “implementation gap”—the question of ‘what does climate adaptation mean in practice?’. The transitions presented earlier (Fig. 1) and the tools and processes developed to enable those transitions were tested in our pilot studies. In this section, we draw on the categories of incremental and transformative change to assess whether we ‘achieved’ the transitions through the Future-Proofing Conservation process, and whether the transitions did indeed accumulate to something that could be regarded as transformative. We conclude with observations around what this project suggests about transformation for climate adaptation theory.

### Did we achieve the transitions?

As noted earlier, our partners were already well advanced on Transition #1, the need to manage by anticipating change. Producing the climate change synthesis report reinforced and refined the knowledge base, and so can be regarded as an incremental contribution. It also, however, played a significant role in advancing the social and political conversation amongst the partners; having this resource gave all participants the confidence to move forward with a clear sense of what is known about possible climate impacts and also what is not known. The uncertainties of what is not known were not debilitating—they opened other conversations about how monitoring may need to be adjusted; but they also framed the broader conversation of what to do, given the state of knowledge. These incremental changes moved participants along Transition #1, but could not answer the question of ‘what does it mean in practice?’

Transition #2 built the foundations for a more critical conversation. The PABAT tool was well received (see Table 2) and readily adopted by PNN. Following our pilot applications, park managers saw immediately that it filled an important role in enabling meaningful dialogue with local communities and stakeholders around the value of the protected areas and integrated it into management planning processes. This was an incremental change, conforming with existing structures. But it also started a conversation about benefits and values being an important role that protected areas played, and so laid the foundations for later steps.

Transition #3, from understanding climate adaptation as a scientific issue to a governance issue, was aided by the knowledge governance interviews. By articulating and reflecting back to participants (many of whom had been interviewed) the ways in which science and other forms of knowledge are used in decision-making, participants recognised that their policymaking, planning, and management were influenced by a range of factors, of which science was only one. This was no surprise of course, but by placing the science-based discourse surrounding climate adaptation in the context of messy, real-world decision-making participants could see the limitations of that framing. They could also relate it to the formal and informal rules that shaped current governance.

The Lessons learned workshop sought to advance Transition #4, from problem-solving to ongoing learning. By identifying lessons from previous activities, examining prior experiences of change and developing a shared language for further deliberation, participants experienced first-hand the insights that could be gained. They were also able to reflect on how dynamic protected areas management is, even though it may feel as though “things never change”. Again, this reiterated and extended existing ideas, representing an incremental shift.

### Did transitions accumulate to transformation?

Each of the prior activities were intended to provide the foundations to enable a more transformative change. While they were all different, they each focused participants’ attention on the connections between the cognitive or conceptual recognition of the need for change, with socio-political aspects of how change may happen. The defining question in this context was whether participants were able to accept and develop the concept of conservation as a matter of preserving benefits from protected areas rather than conserving species, ecosystems or landscape characteristics. This, for us, represented a step that went to the core of the ‘stability’ of conservation practice, challenging the social and political foundation of what protected areas are for. The final activity, the Futures Dialogue, provided a structure for participants to ‘join the dots’ towards this transformation.

There were some indications that the Futures Dialogue was successful in starting such a transformative process. Although it did not achieve clearly transformative actions plans or commitments to change specific policies, it did enable participants to connect their understanding of PA benefits (from the PABAT workshop) with future management goals. The possibility of benefits providing an alternative pathway for conservation when traditional goals of ‘resistance’ (species preservation etc) become unachievable due to climate change was considered by most participants as feasible and attractive. Evaluations conducted immediately post-workshop included comments such as “These 2 days of learning exercise allow us to generate new alternatives for management planning of protected areas” and “This [workshop] has helped feed back and rethink things can we can do as officials, and that we must continue fighting to look at this differently”. (pilot#1). This indicated that participants were able to accumulate smaller transitions in thinking into a larger transformation in how they understand climate adaptation, and what they can do to take action.

### Implications for climate adaption in conservation

The transformation theories that informed this study emphasise the social, institutional and political aspects of decision-making (Wise et al. 2014; Pelling et al. 2015), and the importance of the interaction between cognitive knowledge, social values and institutional rules (Gorddard et al. 2016). On the basis of working directly with partners to address the gap between recognition of the need for change and the implementation of transformation in practice, we found that exploring the social, institutional and political space created options for transforming management that were otherwise not apparent. The study suggests that the cognitive shift of recognising the need for changed management is not difficult; practitioners were already well aware that biophysical change was underway and needed new management strategies. However, the ‘gap’ arose when they sought to apply this cognitive shift directly to policy, planning and management shifts, *without* first examining the social, institutional and political context. The pathway for transformation—for connecting the present with the future—could not extend beyond the incremental *until* change was explored through the lens of existing social, institutional and political arrangements.

In some respects, it may seem counter-intuitive that spending time and resources examining existing governance arrangements creates new options rather than reinforcing them making transformation more difficult to imagine. Yet by reiterating the cognitive shift throughout (“change needs to happen”), and carefully crafting context-specific links between the change in the past and change into the future (“management is dynamic”), the social, institutional and political arrangements opened up new possibilities to bridge the gap. In particular, the idea of benefits providing an alternative pathway for management was a possible transformation that practitioners could understand and relate to. As (Pelling et al. 2015 have written, “Surfacing the full range of adaptation options allows informed questions to be asked of the relationship between adaptation, underlying development priorities and the dominant values that finally determine pathway choice”. The Future-proofing Conservation process enabled participants to surface new options, and understand the values choices that such a transformation would demand.

Other lessons that also emerged from the project offer broader insights into the challenges and opportunities of this collaborative, evolutionary learning-based approach.

#### Resources and limitations

The development and use of this methodology required significant time and resource commitments for the research team, as well as willingness to listen and learn from each other as part of the process. Much has been written about the need to invest in such communication, mediation and translation to support projects at the interface of science, policy and practice (Cornell et al. 2013), and this project was no different. Support from WWF-C and having dedicated staff for coordinating and integrating across partners was critical to meet the challenges of working with complex concepts across an international, multi-sectoral team. While implementing the established process is now less resource-intensive than its development, it still requires resources and capacities that are adequately and realistically matched to project needs.

#### Further application

The Future-Proofing Conservation process was designed to be applied in other protected area contexts. Further piloting to test and refine the concepts will continue. This could result in streamlining the different stages, and tailoring them to different contexts. Application in other locations will still likely require a mix of interdisciplinary academic expertise (social science, climate science, conservation biology) and practical knowledge of people working in protected areas. It is important to recognise that the process can also build capacities in strategic thinking and collective learning; an outcome beyond any action plans created. These broader skills may also facilitate protected areas management processes under uncertain climate variability, incorporating the best scientific knowledge available.

## Conclusions

Conservation has always operated in the context of complex, multifaceted threats. But as climate change-induced ecological transformation becomes a reality, the practical challenges of planning, developing policy and establishing change-ready management practices are emerging. Practitioners and advocates need to develop new ways of articulating the role of conservation as ecosystems change, with new rationales for its importance, and new ideas for achievement. The seeds of these ideas are already well embedded in conservation thinking. Conservationists from all sectors are starting to debate how to value emerging or altered ecosystems; whether and how to intervene in facilitating change; and what kinds of strategies may ensure a more dynamic approach.

The Future-Proofing Conservation process reflects emerging literature (Eriksen et al. 2015; Manuel-Navarrete and Pelling 2015; Wyborn et al. 2016; Colloff et al. 2017) that these debates are largely social and institutional rather than technical, and are intimately connected with how we value natural and cultural landscapes. Adopting the evolutionary learning philosophy brought these values to the fore in an open and consultative way. It allowed conservation participants to consider the future of protected areas in ways that aimed to be empowering and focused on useful ways forward. Co-designing, developing, testing and piloting the activities and ultimately the whole process in active collaboration between academic, civil society and practitioner communities ensured that we struck an appropriate balance between challenging concepts, institutional realities and practical application.

Establishing a shared goal of “future-oriented conservation” is not difficult; the challenge lies in populating this idea with accessible concepts, appropriate strategies and effective practices to bridge the implementation gap. Being able to explore new governance arrangements that anticipate and prepare for ecosystem change while remaining focused on the shared values that underpin protected areas is crucial. By developing this larger transition to new governance, through a series of smaller, interconnected transitions that linked values, rules and knowledge, the participants could work through a series of steps that enable new ways of thinking about the role of protected areas in conservation and consequently new ways to manage them.

## References

[CR1] Abrahms B, DiPietro D, Graffis A, Hollander A (2017). Managing biodiversity under climate change: Challenges, frameworks, and tools for adaptation. Biodiversity and Conservation.

[CR2] Adams RS, Daly SR, Mann LM, Dall’Alba G (2011). Being a professional: Three lenses into design thinking, acting, and being. Design Studies.

[CR3] Adger WN, Dessai S, Goulden M, Hulme M, Lorenzoni I, Nelson DR, Næss LO, Wolf J (2009). Are there social limits to adaptation to climate change?. Climatic Change.

[CR4] Ansell C (2011). Pragmatist democracy: Evolutionary learning as public philosophy.

[CR5] Ansell C, Gash A (2007). Collaborative governance in theory and practice. Journal of Public Administration Research and Theory.

[CR6] Archie KM, Dilling L, Milford JB, Pampel FC (2012). Climate change and western public lands: A Survey of U.S. Federal land managers on the status of adaptation efforts. Ecology and Society.

[CR7] Baron JS, Gunderson L, Allen CD, Fleishman E, McKenzie D, Meyerson LA, Oropeza J, Stephenson N (2009). Options for national parks and reserves for adapting to climate change’. Environmental Management.

[CR8] Beever EA, O’Leary J, Mengelt C, West JM, Julius S, Green N, Magness D, Petes L (2016). Improving conservation outcomes with a new paradigm for understanding species’ fundamental and realized adaptive capacity. Conservation Letters.

[CR9] Beier P, Hansen LJ, Helbrecht L, Behar D (2016). A how-to guide for coproduction of actionable science. Conservation Letters.

[CR10] Berkes F, Colding J, Folke C (2008). Navigating social-ecological systems: Building resilience for complexity and change.

[CR11] Boyd E, Nykvist B, Borgström S, Stacewicz IA (2015). Anticipatory governance for social–ecological resilience. Ambio.

[CR12] Brown T (2008). Design thinking. Harvard Business Review.

[CR13] Chan KMA, Shaw MR, Cameron DR, Underwood EC, Daily GC (2006). Conservation planning for ecosystem services. PLoS Biology.

[CR14] Clark WC, van Kerkhoff L, Lebel L, Gallopin GC (2016). Crafting usable knowledge for sustainable development. Proceedings of the National Academy of Sciences.

[CR15] Colloff MJ, Lavorel S, van Kerkhoff LE, Wyborn CA, Fazey I, Gorddard R, Mace GM, Foden WB (2017). Transforming conservation science and practice for a post-normal world. Conservation Biology.

[CR16] Cornell S, Berkhout F, Tuinstra W, Tàbara JD, Jäger J, Chabay I, de Wit B, Langlais R (2013). Opening up knowledge systems for better responses to global environmental change. Environmental Science & Policy.

[CR18] Dudley N, Stolton S (2009). The protected area benefits assessment tool.

[CR20] Dunlop M (2013). Biodiversity: Strategy conservation. Nature Climate Change..

[CR21] Dunlop M, Parris H, Ryan P, Kroon F (2013). Climate-ready conservation objectives: A scoping study.

[CR22] Eriksen SH, Nightingale AJ, Eakin H (2015). Reframing adaptation: The political nature of climate change adaptation. Global Environmental Change..

[CR23] Figueroa C, Behar K (2017). Análisis de los beneficios de la areas protegidas de la Cuenca Alta del Río Otún.

[CR24] Foden WB, Butchart SHM, Stuart SN, Vié JC, Akçakaya R, Angulo A, DeVantier LM, Gutsche A (2013). Identifying the world’s most climate change vulnerable species: A systematic trait-based assessment of all birds, amphibians and corals. PLoS ONE.

[CR25] Gabriel J (2014). A scientific enquiry into the future. European Journal of Futures Research.

[CR26] Godet M (2006). Creating futures: Scenario planning as a strategic management tool.

[CR27] Gorddard R, Colloff MJ, Wise RM, Ware D, Dunlop M (2016). Values, rules and knowledge: Adaptation as change in the decision context. Environmental Science & Policy.

[CR100] Gross, J., S. Woodley, L.A. Welling, and J.E.M. Watson. 2016. *Adapting to Climate Change: Guidance for protected area managers and planners*. Best Practice Protected Area Guidelines Series No. 24. Gland.

[CR99] Hagerman S, Dowlatabadi H, Chan KMA, Satterfield T (2010). Integrative propositions for adapting conservation policy to the impacts of climate change. Global Environmental Change.

[CR28] Hannah L, Donatti CI, Harvey CA, Alfaro E, Rodriguez DA, Bouroncle C, Castellanos E, Diaz F (2017). Regional modeling of climate change impacts on smallholder agriculture and ecosystems in Central America. Climatic Change.

[CR31] Jantarasami LC, Lawler JJ, Thomas CW (2010). Institutional barriers to climate change adaptation in U.S. national parks and forests. Ecology and Society.

[CR33] Kirchhoff CJ, Carmen Lemos M, Dessai S (2013). Actionable knowledge for environmental decision making: Broadening the usability of climate science. Annual Review of Environment and Resources.

[CR34] Lemieux CJ, Scott DJ (2011). Changing climate, challenging choices: Identifying and evaluating climate change adaptation options for protected areas management in Ontario, Canada. Environmental Management.

[CR35] Lockwood M, Lockwood M, Worboys GL, Kothari A (2006). Values and benefits. Managing protected areas: A global guide.

[CR37] Lonsdale WR, Kretser HE, Chetkiewicz CB, Cross MS (2017). Similarities and differences in barriers and opportunities affecting climate change adaptation action in four north american landscapes. Environmental Management.

[CR38] Manuel-Navarrete D, Pelling M (2015). Subjectivity and the politics of transformation in response to development and environmental change. Global Environmental Change.

[CR39] Mauser W, Klepper G, Rice M, Schmalzbauer BS, Hackmann H, Leemans R, Moore H (2013). Transdisciplinary global change research: The co-creation of knowledge for sustainability. Current Opinion in Environmental Sustainability.

[CR40] Metcalf SJ, van Putten EI, Frusher SD, Marshall NA, Tull M, Caputi N, Haward M, Hobday AJ (2015). Measuring the vulnerability of marine social–ecological systems: A prerequisite for the identification of climate change adaptations. Ecology and Society.

[CR98] Olsson, P., L.H. Gunderson, S.R. Carpenter, P. Ryan, L. Lebel, C. Folke, and C.S. Holling. 2006. Shooting the rapids: Navigating transitions to adaptive governance of social-ecological systems. *Ecology and Society* 11(1).

[CR41] Ostrom E (1999). Coping with the tragedy of the commons. Annual Review of Political Science.

[CR42] Pelling M (2011). Adaptation to climate change: From resilience to transformation, adaptation to climate change: from resilience to transformation.

[CR43] Pelling M, O’Brien K, Matyas D (2015). Adaptation and transformation. Climatic Change.

[CR45] Rannow S, Macgregor NA, Albrecht J, Crick HQP, Förster M, Heiland S, Janauer G, Morecroft MD (2014). Managing protected areas under climate change: Challenges and priorities. Environmental Management.

[CR46] Stein BA, Staudt A, Cross MS, Dubois NS, Enquist C, Griffis R, Hansen LJ, Hellmann JJ (2013). Preparing for and managing change: Climate adaptation for biodiversity and ecosystems. Frontiers in Ecology and the Environment.

[CR47] Tanner-McAllister SL, Rhodes J, Hockings M (2017). Managing for climate change on protected areas: An adaptive management decision making framework. Journal of Environmental Management.

[CR48] Tschakert P, Dietrich KA (2010). Anticipatory learning for climate change adaptation and resilience. Ecology and Society.

[CR50] van Kerkhoff L, Pilbeam V (2017). Understanding socio-cultural dimensions of environmental decision-making: A knowledge governance approach. Environmental Science & Policy.

[CR51] Vogel, I. 2012. Review of the use of ‘Theory of Change’ in international development. Report for DFID. UK. https://assets.publishing.service.gov.uk/media/57a08a5ded915d3cfd00071a/DFID_ToC_Review_VogelV7.pdf. Accessed 20 April 2016.

[CR53] Wise RM, Fazey I, Stafford Smith M, Park SE, Eakin HC, Archer Van Garderen ERM, Campbell B (2014). Reconceptualising adaptation to climate change as part of pathways of change and response. Global Environmental Change.

[CR95] West JM, Julius SH, Kareiva P, Enquist C, Lawler JJ, Petersen B, Johnson AE, Shaw MR (2009). U.S. natural resources and climate change: Concepts and approaches for management adaptation. Environmental Management.

[CR52] Wyborn C, van Kerkhoff L, Dunlop M, Dudley N, Guevara O (2016). Future oriented conservation: Knowledge governance, uncertainty and learning. Biodiversity and Conservation.

